# A rapid turnaround gene panel for severe autoinflammation: Genetic results within 48 hours

**DOI:** 10.3389/fimmu.2022.998967

**Published:** 2022-09-20

**Authors:** Dara McCreary, Ebun Omoyinmi, Ying Hong, Barbara Jensen, Alice Burleigh, Fiona Price-Kuehne, Kimberly Gilmour, Despina Eleftheriou, Paul Brogan

**Affiliations:** ^1^ Inflammation and Rheumatology Section, University College London Great Ormond Street Institute of Child Health, London, United Kingdom; ^2^ National Amyloidosis Centre, Royal Free Hospital, London, United Kingdom; ^3^ Centre for Adolescent Rheumatology, University College London, London, United Kingdom; ^4^ Camelia Botnar Laboratory, Great Ormond Street Hospital National Health Service (NHS) Foundation Trust, London, United Kingdom; ^5^ Rheumatology Department, Great Ormond Street Hospital National Health Service (NHS) Foundation Trust, London, United Kingdom

**Keywords:** genetics, autoinflammation, immunodeficiency, hyper-inflammation, turnaround time (TAT)

## Abstract

There is an important unmet clinical need for fast turnaround next generation sequencing (NGS) to aid genetic diagnosis of patients with acute and sometimes catastrophic inflammatory presentations. This is imperative for patients who require precise and targeted treatment to prevent irreparable organ damage or even death. Acute and severe hyper- inflammation may be caused by primary immunodeficiency (PID) with immune dysregulation, or more typical autoinflammatory diseases in the absence of obvious immunodeficiency. Infectious triggers may be present in either immunodeficiency or autoinflammation. We compiled a list of 25 genes causing monogenetic immunological diseases that are notorious for their acute first presentation with fulminant inflammation and which may be amenable to specific treatment, including hemophagocytic lymphohistiocytosis (HLH); and autoinflammatory diseases that can present with early-onset stroke or other irreversible neurological inflammatory complications. We designed and validated a pipeline that enabled return of clinically actionable results in hours rather than weeks: the Rapid Autoinflammation Panel (RAP). We demonstrated accuracy of this new pipeline, with 100% sensitivity and 100% specificity. Return of results to clinicians was achieved within 48-hours from receiving the patient’s blood or saliva sample. This approach demonstrates the potential significant diagnostic impact of NGS in acute medicine to facilitate precision medicine and save “life or limb” in these critical situations.

## Introduction

In recent years there has been increased adoption of next generation sequencing (NGS) to facilitate molecular diagnoses and routine clinical care. The impact of this has been particularly significant for patients with immunological diseases, notably those with primary immune deficiency (PID) or autoinflammation (1–3). NGS has facilitated the discovery and classification of many new immunological diseases, sometimes providing insights into pathogenesis and resulting in discovery and implementation of targeted treatments (4). A major challenge for the implementation of NGS into routine clinical practice, however, is the time it takes to receive results. Availability of NGS remains patchy within individual countries and throughout Europe, a challenge that the European reference network for rare immunological and autoinflammatory diseases (ERN-RITA) seeks to address (5); but even in countries and centers where this is available, turnaround time (TAT) is usually weeks to months, rather than hours to days. For instance, in the South-East of England, TAT for a modest (24-gene) autoinflammatory panel is 84 days from receipt of a DNA sample. Additional delays incurred by extraction of DNA and transportation to the central genomics laboratory hub may add a further 2-4 weeks. Clinicians, and most importantly patients with chronic inflammatory diseases, are increasingly frustrated by this slow TAT; an issue that becomes especially critical for patients with acute fulminant hyper-inflammatory presentations, who may accrue irreparable organ injury, significant glucocorticoid toxicity (6, 7), or even die during the pre-diagnostic phase of their illness.

The Great Ormond Street Hospital Autoinflammation Centre of Excellence (GOSH-ACE) receives acute referrals nationally in the UK, and internationally. We increasingly noted a crucial unmet clinical need in our service regarding slow TAT of genetic results for acutely unwell pediatric patients. This particularly related to patients presenting for the first time with acute hyper-inflammation, and included various immunological diseases such as hemophagocytic lymphohistiocytosis (HLH) (6, 8, 9); and autoinflammatory diseases presenting with acute vasculitic features such as arterial stroke, digital gangrene, or other life and organ threatening vasculitic injury (e.g. deficiency of adenosine deaminase 2, DADA2; or STING associated vasculopathy with onset in infancy, SAVI, amongst several others). We concluded that there was a need for a bespoke NGS targeted gene panel that would screen for a select number of genetic immunological diseases that in our experience would be important to identify acutely, since targeted treatment might be available.

Previously our group had designed and implemented two diagnostic targeted gene panels which we now deploy for routine patient care: the vasculitis and inflammation panel (2); and the neuroinflammation panel (1). We hypothesized that we could adapt these pipelines to achieve a TAT of 48 to 72 hours. We defined TAT as the time from receipt of whole blood or saliva to the delivery of a formal written report to the clinician. We included 25 monogenetic immunological diseases (the rapid autoinflammation panel, RAP) that in our experience could present acutely with fulminant hyper-inflammation (with or without infection), where rapid deployment of specific or targeted treatment might improve the prognosis (**Table 1**). The aims of this study were therefore to: 1. design a pathway with potential TAT of 48-72h; 2. assess the accuracy of the readout in healthy controls, and disease controls with known genetic diagnoses; and 3. apply this to consecutive newly presenting patients referred to our service to ascertain the feasibility of this new pipeline in an acute clinical setting.

**Table 1:** The Rapid Autoinflammation Panel (RAP).

Gene ID	Gene name	Disease	Prevalence(*Orphanet)	Potential acute presentation	Specific/targeted treatment to be considered
***ADA***	Adenosine deaminase	SCID	1-9/1,000,000	Sepsis; lymphopenia	Allo-HSCT; gene therapy; enzyme replacement therapy
***ADA2***	Adenosine deaminase 2	DADA2	<1/1,000,000	Stroke or non-CNS vasculitic emergency	Anti-TNF
***ADAR1***	Adenosine deaminase, RNA specific	AGS type 6	Unknown; >120 cases reported	Early-onset encephalopathy, cardiac calcification	JAKI
***DCLRE1C***	DNA cross-link repair 1C	SCID Omenn syndrome	Unknown	Sepsis; lymphopenia erythrodermatitis	Allo-HSCT
***IFIH1***	Interferon induced with helicase C domain 1	AGS type 7	Unknown; >120 cases reported	Early-onset encephalopathy, cardiac calcification	JAKI
***IL2RG***	Interleukin 2 receptor, gamma	SCID	1/200,000	Sepsis; lymphopenia	Allo-HSCT; gene therapy
***LYST***	Lysosomal trafficking regulator	HLH Chedak-Higashi disease	Unknown; <500 cases reported	HLH	Allo-HSCT
***NLRC4***	NLR family card domain containing 4	AIFEC FCAS4	Unknown	MAS; fulminant enterocolitis	GC, IL-1 blockade, anti-TNF; IL-18BP**
***PRF1***	Perforin 1	Familial HLH type 2	<1/1,000,000	HLH	Allo-HSCT
***RAB27A***	RAB27A, member RAS oncogene family	HLH Gricelli syndrome	Unknown	HLH	Allo-HSCT
***RAG1***	Recombination activating gene 1	SCID Omenn syndrome	Unknown	Sepsis; lymphopenia; erythrodermatitis	Allo-HSCT; gene therapy
***RAG2***	Recombination activating gene 2	SCID Omenn syndrome	Unknown; >120 cases reported	Sepsis; lymphopenia erythrodermatitis	Allo-HSCT
***RNASEH2A***	Ribonuclease H2 subunit A	AGS type 4	Unknown; >120 cases reported	Early-onset encephalopathy	JAKI
***RNASEH2B***	Ribonuclease H2 subunit B	AGS type 2	Unknown; >120 cases reported	Early-onset encephalopathy	JAKI
***RNASEH2C***	Ribonuclease H2 subunit C	AGS type 3	Unknown; >120 cases reported	Early-onset encephalopathy	JAKI
***SAMHD1***	SAM and HD domain containing deoxynucleoside triphosphate triphospho-hydrolase 1	AGS type 5	Unknown; >120 cases reported	Early-onset encephalopathy	JAKI
***SH2D1A***	SH2 domain containing 1A	HLH/XLP	<1/1,000,000	HLH	Allo-HSCT
***SLC29A3***	Solute carrier family 29 member 3	HLH	<1/1,000,000	HLH	Allo-HSCT
***STX11***	Syntaxin 11	Familial HLH type 4	Unknown	HLH	Allo-HSCT
***STXBP2***	Syntaxin binding protein 2	Familial HLH type 5	Unknown	HLH	Allo-HSCT
***STING1***	Transmembrane protein 173	SAVI	<1/1,000,000	Vasculitic ischemic skin lesions; interstitial lung disease	GC; JAKI
***TREX1***	Three prime repair exonuclease 1	AGS type 1	Unknown; >120 cases reported	Early-onset encephalopathy	JAKI
***UNC13D***	UNC-13 homolog D	Familial HLH type 3	Unknown	HLH	Allo-HSCT
***WAS***	Wiskott-Aldrich syndrome	PID/WAS	1-9/1,000,000	Systemic inflammation, enterocolitis, thrombocytopenia	Allo-HSCT; gene therapy; supportive treatment
***XIAP***	X-linked inhibitor of apoptosis	PID/HLH (XLP2)	<1/1,000,000	HLH; colitis; hypogammaglobulinemia; sepsis; hepatosplenomegaly	Allo-HSCT

RAP contains 25 genes causing diseases that may present acutely, and for which specific or targeted treatments are available. *Orphanet: https://www.orpha.net/consor/cgi-bin/index.php, **IL-18BP, interleukin 18 binding protein (investigational treatment). SCID, severe combined immunodeficiency; allo-HSCT, allogeneic hematopoietic stem cell transplantation; DADA2, deficiency of adenosine deaminase 2; CNS, central nervous system; anti-TNF, anti-tumor necrosis factor; AGS, Aicardi Goutières syndrome; JAKI, janus-associated kinase inhibitors; SCID, severe combined immunodeficiency; HLH, hemophagocytic lymphohistiocytosis; AIFEC, autoinflammation with infantile enterocolitis; SAVI, STING-associated vasculopathy with onset in infancy; FCAS4, familial cold autoinflammatory syndrome type 4; MAS, macrophage activation syndrome; GC, glucocorticoids; IL-1, interleukin-1; PID, primary immunodeficiency; WAS, Wiskott-Aldrich syndrome; XLP2, X-linked lymphoproliferative disease 2.

## Materials and methods

### Ethical approval

This study was approved by the National Research Ethics Service Committee (research ethics number 08H071382). All participants and parents provided written consent or assent as appropriate.

### Controls

We recruited patients referred to our tertiary referral center (GOSH-ACE). Patients with known genotypes (n=15) from our database served as disease controls to assess accuracy of the new panel. We included 14 samples from patients who were known to have a variant in at least one of the genes included in the RAP; and a single patient with acute disseminated encephalomyelitis (ADEM) known to be without any such variants (**Supplemental Table 1**). These variants had previously been identified through either Sanger sequencing or NGS performed as part of their routine care. Genome in a Bottle, a DNA reference material, was also used to further evaluate the performance of the RAP, as described previously (10, 11). We sequenced the Genome in a Bottle sample 4 times on 3 separate panel runs to confirm adequate capture of all regions in the panel designed (data not shown).

### Prospective patients

Genetic sequencing using the RAP was undertaken in 15 consecutive cases presenting acutely between December 1, 2019, and December 22, 2021, where there was clinical concern for a genetic cause of disease. For each case, in addition to the RAP pipeline, we concurrently sequenced each case using our validated routine larger targeted panels, modified over time to include new genes discovered since we originally published on these panels (1, 2) as the clinical gold-standard used to check the accuracy of the RAP. **Table 2** summarizes the clinical indication for requesting genetic testing.

**Table 2:** Prospective patients, clinical indication for requesting the RAP and final diagnosis.

PatientAge (years)	Clinical indication for requesting the rapid autoinflammation panel (RAP)	*Rare genetic variants identified by RAP		Clinical impact of RAP	Concordance with routine NGS panels (VIP or NIP)	**Final clinical diagnosis
		Amino acid change	Zygosity			
**Patient 1** **4y**	Ischaemic stroke, CT scan showed bilateral anterior cerebral artery vasculopathy and bifrontal subcortical calcification, ESR>150mm/hr (reference range 0-10mm/hr); exclude DADA2 or other genetic neuroinflammatory disorder	*ADA* p.R142Q	Het	DADA2 excluded; no genotype to explain phenotype	Confirmed with NIP	Idiopathic arterial stroke
**Patient 2** **7y**	Pustular autoinflammation, autism; exclude monogenetic autoinflammation	*DCLRE1C* p.E632K	Het	No genotype to explain phenotype	Confirmed with VIP	Idiopathic neutrophilic dermatosis (resolved)
		*PRF1* p.A211V	Het	
		STX11 p.T74M	Het	
**Patient 3** **5y**	Respiratory failure secondary to progressive fibrotic interstitial lung disease, skin rashes, anti-MDA5 positive, family history recurrent infections.	*PRF1* p.A211V	Het	Possible hypomorphic RAG deficient SCID	Confirmed with NIP	SCID not confirmed. JDM with ILD likely final diagnosis; patient deceased
		*RAG1* p.E193K	Het	
		*RAG1* p.R449K	Het	
		*RAG2* p.F386L	Het	
		***RAG2*** **p.T215I**	Het	
**Patient 4** **15y**	Intracranial hemorrhage, refractory immune thrombocytopenia: exclude WAS or neuroinflammatory	***PRF1*** **p.A91V**	Het	WAS excluded; no genotype to explain phenotype	Confirmed with NIP	Immune thrombo-cytopenia with intracranial hemorrhage
**Patient 5** **0.5y**	Unexplained neonatal inflammation; exclude HLH	*LYST* p.R2624W	Het	Familial HLH excluded	Confirmed with VIP	Autoinflammation of unknown cause
		***PRF1*** **p.A91V**	Het	
		*WAS* p.P460S	Het	
**Patient 6** **12y**	Chronic EBV viraemia, indeterminate intestinal inflammation, alopecia, hepatic failure; exclude primary HLH prior to liver transplantation	*RNASEH2B* p.L84V	Het	Familial HLH excluded	Confirmed with large immunology panel	Autoimmune hepatitis type I
		*XIAP* p.P218L	Het	
		*IFIH1* p.P132S	Het	
**Patient 7** **1y**	Unexplained neonatal inflammation, anemia, thrombocytopenia; exclude HLH	*RNASEH2A* p.K221R	Het	HLH excluded; no genotype to explain phenotype	Confirmed with VIP	Autoinflammation of unknown cause
**Patient 8** **8y**	Extensive unexplained acute cutaneous infarction; exclude DADA2	–	–	DADA2 excluded; no genotype to explain phenotype	Confirmed with VIP	Purpura fulminans (no infectious cause found)
**Patient 9** **1y**	Atypical Kawasaki disease with sustained, chronic inflammation, widespread nodular rash and nail dystrophy; exclude autoinflammatory disease	*LYST* p.R2050Q	Het	No genotype to explain phenotype	Confirmed with VIP	Kawasaki disease
**Patient 10** **18y**	Probable adult-onset Still’s disease and MAS; exclude primary HLH	–	–	HLH excluded	Confirmed with VIP	Adult-onset Still’s disease and MAS
**Patient 11** **1y**	Probable systemic onset JIA, strong family history of deafness; exclude monogenetic autoinflammation	*NLRC4* p.A929S	Het	No genotype to explain phenotype	Confirmed with VIP	Systemic onset JIA
**Patient 12** **9y**	Recurrent fevers with aphthous ulcers, lymphadenopathy, pharyngitis and abdominal pain; exclude SAVI	*DCLRE1C* c.G112A	Het	No genotype to explain phenotype	Confirmed with VIP	SURFS
**Patient 13** **1y**	Suspected MAS, recurrent atypical febrile seizures, respiratory failure with episodic cardiac tachyarrhythmias; exclude primary HLH	*DCLRE1C* p.S635Kfs*5	Het	HLH excluded; no genotype to explain phenotype	Confirmed with VIP	Suspected Catecholaminergic polymorphic ventricular tachycardia syndrome (CPVT); patient deceased
**Patient 14** **11y**	Idiopathic arterial stroke; exclude DADA2	*LYST* p.D3289N	Het	DADA2 excluded; no genotype to explain the phenotype	Confirmed with VIP	Primary angiitis of the central nervous system (PACNS)
		***PRF1*** **p.A91V**	Het	
**Patient 15** **13y**	Takayasu arteritis and neuroinflammation with intracranial vasculopathy; exclude monogenetic neuroinflammatory	*LYST* p.S1840A	Het	No genotype to explain phenotype	Confirmed with NIP	Takayasu arteritis complicated by cerebral vasculitis
		*IFIH1* p.K349R	Het	

*Class 4 or 5 genetic variants (pathogenic mutations) highlighted in bold and note that PRF1 p.A91V has minor allele frequency 2-9%. **Final diagnosis after consideration of full clinical work-up, including routine clinical genetic testing using whole exome sequencing or routine larger NGS panels (either VIP, n= 10 cases; or NIP, n= 4 cases). NGS, next generation sequencing; VIP, vasculitis and inflammation gene panel; NIP, neuroinflammation gene panel; ESR, erythrocyte sedimentation rate; CT, computed tomography; het, heterozygous; hom, homozygous; DADA2, deficiency of adenosine deaminase 2; anti-MDA5, anti-melanoma differentiation-associated gene 5,JDM, juvenile dermatomyositis; RAG, recombination activating gene; SCID, severe combined immunodeficiency; ILD, interstitial lung disease; WAS, Wiskott-Aldrich syndrome; HLH, hemophagocytic lymphohistiocytosis; EBV, Epstein-Barr virus; MAS, macrophage activation syndrome; JIA, juvenile idiopathic arthritis; SAVI, STING associated vasculopathy with onset in infancy; SURFS, systemic undifferentiated recurrent fever syndrome; MAS, macrophage activation syndrome.

### Workflow design for the RAP

**Figure 1:**
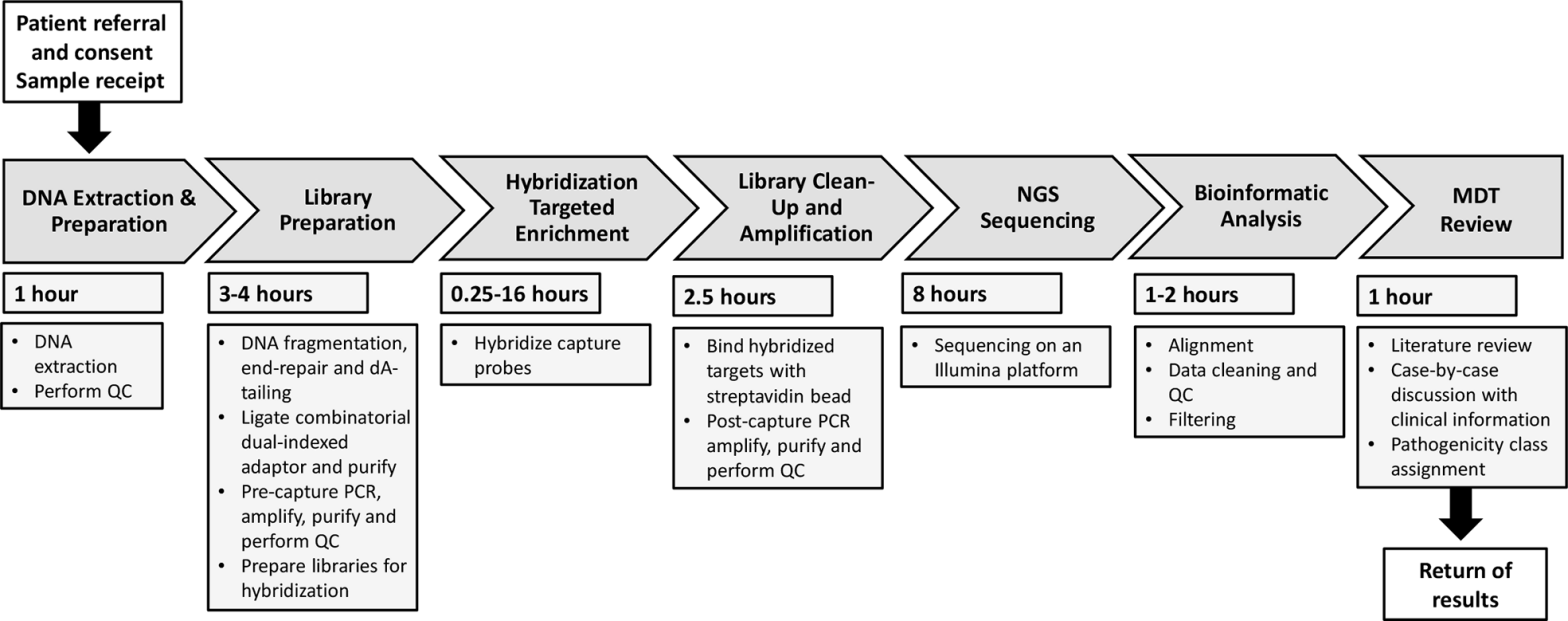
Overall workflow for the Rapid Autoinflammation Panel (RAP). This flow diagram represents the seven stages of the RAP workflow demonstrating how return of results is achieved <48 hours from receiving a patient sample. QC, quality control; PCR, polymerase chain reaction; NGS, next-generation sequencing; MDT, multidisciplinary team.

We considered each component step of the workflow sequence from first contact from the requesting clinician, to return of formal genetic report. We next determined the fastest feasible time to complete each step, to achieve a TAT of within 48h, and allocated resources as required to achieve that, as summarized in **Figure 1**.

### DNA preparation

Upon receipt of the request (by email) from the clinician, we immediately arranged obtaining a sample for extraction of DNA. We extracted DNA from either blood (EDTA sample) or saliva. One of the most critical time-saving steps was getting the sample to the research team as quickly as possible, achieved by a combination of direct delivery from the clinician, or direct collection by the researcher (DM). DNA was extracted from the EDTA blood sample using the Gentra Puregene extraction kit (Qiagen); DNA from saliva was extracted using the prepIT L2P kit (Genotek).

### Library preparation and targeted enrichment

The design and development of the capture library was done with Twist Bioscience (https://www.twistbioscience.com) and was designed to work with the Illumina sequencing kit. The library capture was performed as described in the Twist Bioscience library preparation protocol. First, enzymatic fragmentation was performed before undergoing the Twist Universal Adaptor System step. This was then followed by the Twist Fast Hybridization Target Enrichment Protocol. Once the DNA was extracted it underwent quality control (QC) and was then diluted to 5ng/μl prior to starting the fragmentation. Briefly, the genomic DNA was fragmented by enzymatic reaction, then the fragments end-repaired and dA-tailed. The Twist universal adaptors were then ligated to the dA-tailed DNA fragments before purifying to generate indexed gDNA libraries. The indexed gDNA library was amplified and purified before the QC of the sample was performed. This was performed using TapeStation 4200 Bioanalyzer (Agilent) to assess the fragment size of the indexed library.

### Library clean-up and amplification

The indexed ligated libraries were amplified and hybridized to our customized Twist gene panel (**Table 1**) before being amplified *via* polymerase chain reaction (PCR). The protocol enabled samples to be processed as a single sample, or multiplex. The hybridization step was incubated for a minimum of 15 minutes; or left to run overnight (depending on the time of receiving the patient sample and the urgency of results). Once the hybridization was complete, the samples were cleaned using purification beads before undergoing a final amplification step.

### Sequencing

The captured and indexed library was then sequenced as a single sample using the Illumina MiSeq sequencer with either MiSeq Reagent Kit v2 (500-cycles) MS-102-2003 or the Micro Kit V2 (300-cycles) MS-103-1002. A region was considered a low-coverage region if any single nucleotide in the exon had a coverage less than 30x. After the first eight samples were sequenced, additional baits were added to a total of 8 regions to improve coverage of 4 genes. Subsequently, overall coverage was high and reproducible between the different sequencing runs.

### Bioinformatics

Read alignment, variant calling, and annotation were performed using the web-based Galaxy (12). The method uses paired end reads from Illumina MiSeq instruments that were mapped to the human reference genome (GRCh37) using Burrows-Wheeler Aligner–MEM software. The output variant call format (VCF) file from USEGALAXY was annotated using wANNOVAR, the web-based user-interfaced ANNOVAR tool from Wang Genomic Labs, which provided allele frequencies from public databases and in-silico predictions of pathogenicity (13). Identified variants were evaluated for coverage using the Integrative Genomics Viewer (14). We used public databases to search for the frequency of variants: the 1000 Genome Project, Exome Variant Server, Exome Aggregation Consortium, and Genome Aggregation database (15–18). We filtered out synonymous variants and then excluded common polymorphic variants with minor allele frequency of 1% or greater. The only exception to this was the relatively common pathogenic *PRF1* p.Ala91Val variant (19). Pathogenicity assessment of identified variants was done from the downloaded wANNOVAR files, and predicted functional impact of any variants using SIFT, PolyPhen-2 and MutationTaster (20–22).

### Report writing and return of results

The final step in the RAP workflow was a formal multidisciplinary team (MDT) reporting meeting involving clinicians (PB and DE) and scientists (DM and EO). At that meeting, the clinical phenotype was reviewed in the context of any rare genetic variants identified and a literature review was conducted to inform the pathogenicity assessment. The identified variants were individually assessed and classified into pathogenicity groups (class 1, clearly not pathogenic; class 2, unlikely to be pathogenic; class 3, unknown significance; class 4, likely to be pathogenic; and class 5, clearly pathogenic) according to the Association for Clinical Genetic Science 2013 practice guidelines (23). Each meeting typically took between 30 and 60 minutes per patient. Signed return of results reports were then emailed securely to the lead clinician caring for the patient within 48 hours of sample receipt.

### Statistical analysis

Continuous variables were summarized as median and range. Categorical variables were presented as percentages and frequencies. Sensitivity and specificity were calculated using SPSS statistical software version 21 (IBM).

## Results

### Turnaround time for the RAP

The time taken from receipt of sample for DNA separation to return of report for the 15 prospective cases studied was under 48 hours, confirming our methodological workflow time-based estimate.

### Accuracy of the RAP in disease controls

The RAP correctly identified all 16 variants known to be present in the 15 disease controls shown in **Supplemental Table 1** and did not falsely call any other rare variants. One disease control sample was known to have no rare variants in any of the genes covered and served as a negative control; this was also confirmed by the RAP. Thus, the RAP was both highly sensitive (100%) and specific (100%) for detection of these known rare variants. In addition, two of the disease control samples *ADA2* (p.G47R) and *ADA2* (p.P251L) were run on separate runs to check reproducibility of the pipeline, with 100% concordance (data not shown).

### Performance of the RAP in prospective patients with unknown diagnoses

Between December 1, 2020, and December 22, 2021, the RAP was requested to be performed on 15 patients referred to our service who required urgent diagnostic genetic testing; 10 males; median age, 7.2 years (range 0.5-18 years). The RAP had actionable clinical impact in all 15 patients by excluding the specific genetic cause queried by the clinician in 14/15 cases, and in one case (patient 3) the RAP identified a non-confirmatory genotype suggestive of hypomorphic recombination-activating gene (RAG) severe combined immunodeficiency (SCID). A total of 25 variants (22 rare variants and the three PRF1 variants) were identified in these 15 patients (median 1, range 0-5 variants per patient). A summary of the phenotypes, genotypes, and final diagnoses of these 15 patients is provided in **Table 2**.

### Pathogenic variants (Class 4 or Class 5)

Class 4 or 5 pathogenic variants are highlighted in bold in **Table 2**. Patient 3 was a 5-year-old boy from consanguineous parents of Bangladeshi origin. There was a positive family history of a paternal uncle who died of infection at age 3; and a second paternal uncle with recurrent infections now aged 35 years. The proband had two younger siblings with no significant past medical history. He was admitted to the pediatric intensive care unit with a 4-to-6-week history of progressive respiratory failure, with negative infection screen for SARS-CoV-2. Cutaneous features were reminiscent of juvenile dermatomyositis (JDM); and anti-melanoma differentiation associated gene 5 (MDA5) antibodies were positive. Cytomegalovirus (CMV) viremia and CMV were detected in bronchoalveolar lavage raising the possibility of CMV pneumonitis as a contributing cause of the respiratory failure. Immunophenotyping demonstrated low T, normal B, almost absent NK cells, and low naïve CD4 cells. Serum immunoglobulin levels were normal. The RAP identified 2 rare variants in *RAG1;* 2 variants in *RAG2;* and a single *PRF1* variant **(** **Table 2** **)**. These results suggested a possible (but unconfirmed) diagnosis of severe combined immunodeficiency (SCID). The *RAG2* (p.T215I) variant has been described previously and associated with typical SCID (24). The *RAG2* (p.F386L) variant results in wild-type activity (National Institute of Health, personal communication, data not shown) and is therefore likely benign; both *RAG1* variants were also classified as benign. All other common SCID mutations were excluded in this case using our wider genetic panels (*IL2RG*, *JAK3*, *RAG1*, *DCLRE1C*, *PRKDC*, *LIG4*, *NHEJ1*, *ADA*, *AK2*, *IL7R*) (1). Carrier status for the *PRF1* (p.A211V) variant was an incidental finding. The patient died, thus precluding further immunophenotyping for hypomorphic RAG deficient SCID. We concluded that the most likely diagnosis was that of JDM with interstitial lung disease as the cause of death. Patient 4, 5 and 14 (**Table 2**) were carriers for *PRF1* (p.A91V) (19), again deemed incidental to the phenotype.

### Variants of unknown significance (Class 3)

A total of 21 unique variants of unknown significance (VUS) in 12 genes were found in 13 patients. Two patients (13.3%) were found to have no rare variants in any of the 25 genes covered in the RAP (**Table 2** **)**.

## Discussion

The diagnostic utility of next-generation sequencing (NGS) has undoubtedly transformed medicine in virtually every specialty. A major limitation, however, is the turnaround time (TAT) for clinically actionable results. Increasingly, molecular diagnoses guide therapeutic decisions, and these decisions are particularly critical for acutely sick patients presenting with suspected monogenetic immunological diseases (4, 6). In this study, we designed and validated a modest targeted panel to aid the diagnosis of patients presenting with fulminant inflammation. We based the choice of the 25 genes included in the RAP (**Table 1**) purely on the needs of our service as a tertiary referral center for autoinflammation, based on our collective clinical experience of caring for patients with autoinflammation or immunodeficiency. We devised and delivered a workflow that enabled a TAT of less than 48 hours, defined as the time taken from receipt of a sample for DNA extraction to return of an actionable report to the clinician. We validated the accuracy of RAP by comparing it to our routine clinical NGS pipelines and found 100% concordance with these established pipelines.

We found that there were three major resource requirements required to achieve a TAT of less than 48 hours: 1. a dedicated skilled scientist to deliver the panel, including collecting samples from the requesting clinician in real time; 2. access to a sequencer in real time with no notice or need for prior booking; and 3. availability of skilled personnel (including scientists and clinician scientists) to deliver a multidisciplinary team (MDT) report. In summary, these results suggest that it is possible to deliver NGS genetic test results within 48 hours if there is adequate skilled manpower, and availability of a sequencer. The choice of genes included on such rapid return panels can be modified for different clinical settings.

The Genomic Medicine Service (GMS) within the NHS in England (https://www.england.nhs.uk/publication/national-genomic-test-directories/) offers a rapid sequencing service for acutely unwell babies and children with a potentially monogenic disorder, initially applying a targeted virtual gene panel to whole exome/genome sequencing data, with a TAT of 14 days for negative/preliminary results; and 21 days for a final report confirming a clinically relevant finding. Other laudable attempts to shorten the TAT for results include a Canadian study with mean time to preliminary report of 7.2 days (25); pilot studies in the US achieving a mean TAT between 14 and 16.3 days for a formal written report (26, 27); and a Chinese group that managed to deliver a 24-hour rapid trio exome in critically unwell children (28). However, these studies were in research settings, and have not yet translated into routine clinical care. Perhaps more relevant to our study was a recent report from Australia that demonstrated the feasibility of ultra-rapid exome sequencing in a public healthcare system, achieving a median of 3 days from sample receipt to clinical report (29). The cost of this service, however, was not described.

We recognize that rapid return of whole exome/genome sequencing results most likely not be achieved for years to come, although as the cost and speed of NGS continues to decline, we anticipate this will be available in the foreseeable future. However, until this is achieved, panel sequencing remains the mainstay for rapid genetic testing in a clinical setting. The RAP was not designed to replace larger gene panel or clinical whole-exome sequencing that are increasingly part of routine clinical care. Rather, it is designed to be implemented at the beginning of the diagnostic workup for patients with severe inflammatory presentations. We anticipate that modifications of the selection of the genes may be required to further optimize the diagnostic yield. It is unsurprising that none of the 15 prospective patients we studied turned out to conclusively have any of the genetic diseases included in the RAP (nor were any genetic diagnoses identified with subsequent larger panel or WES in these patients). This does not deter from the clinical impact, since a negative test result using a test with a very high negative predictive value is very useful for clinicians, as indicated in **Table 2** for each of the 15 patients we studied. Clinicians are increasingly aware that genetic testing for ultra-rare diseases usually returns negative results, and that this allows rapid exclusion of these rare but treatable diseases early in the patient pathway (30).

Several practical factors can adversely affect the TAT. Lessons learned to mitigate against barriers to rapid TAT include the aforementioned availability of a dedicated and skilled workforce; and open access to a sequencer. Other practical considerations are co-location of the sequencing laboratory and personnel close to the requesting clinical site; clear lines of communication between requesting clinicians and scientists; and close working relationships between scientists and clinician scientists to generate reports. Consideration of maintaining patient confidentiality must always be remembered when returning reports to clinicians *via* email. Another practical consideration regarding implementation is cost; in the UK, the current cost of a gene panel is in the region of £750 to £1030 (National Health Service North Thames genomics laboratory Hub, personal communication). An estimate of the cost of the RAP per patient (excluding the potentially expensive input of the MDT to generate reports) was approximately £1000.

While targeted gene panels can also offer benefit for detecting somatic mutations, they must be designed for this purpose, i.e., careful selection of a restricted number of genes and ensuring high read depth by using a sequencing technology with greater output. Our study chose a read depth of a minimum of 30x, which is commonly accepted for diagnostic purposes (31), but higher coverage would be required for detection of somatic mutations. Furthermore, somatic mutations in the 25 RAP genes we included are not known to cause disease at low levels of somatic mosaicism.

In conclusion, we have devised and validated a workflow that successfully offers rapid return of genetic test results within 48 hours. The panel was designed to aid the diagnosis of patients presenting with fulminant inflammation but could be modified for virtually any clinical setting where rapid return of results may modify treatment and improve patient outcomes. The main resources required to deliver this workflow included a dedicated skilled scientist, open access to a sequencer, and availability of a rapid response skilled clinical/scientific MDT to generate reports. We suggest that our study brings us ever closer to the ultimate desirable goal of rapid return of genetic results at the point of patient care.

## Data availability statement

The original contributions presented in the study are included in the article/**Supplementary Material**. Further inquiries can be directed to the corresponding author.

## Ethics statement

The studies involving human participants were reviewed and approved by National Research Ethics Committee: research ethics number 08H071382. Written informed consent to participate in this study was provided by the participants’ legal guardian/next of kin.

## Author contributions

DM, EO, DE, KG, and PB conceptualized the study; DM, FP-K, and PB wrote the first draft of the manuscript; DM, EO, AB, YH, BJ and FP-K collected and analyzed the data. All authors contributed to the final article and approved the submitted version.

## Funding

This study was funded in whole from a peer-reviewed grant from Rosetrees Trust (grant number CF1\100009). All research at Great Ormond Street Hospital NHS Foundation Trust and UCL Great Ormond Street Institute of Child Health is supported by the NIHR Great Ormond Street Hospital Biomedical Research Centre.

## Acknowledgments

We thank all families who agreed to take part in this study.

## Conflict of interest

The authors declare that the research was conducted in the absence of any commercial or financial relationships that could be construed as a potential conflict of interest.

## Publisher’s note

All claims expressed in this article are solely those of the authors and do not necessarily represent those of their affiliated organizations, or those of the publisher, the editors and the reviewers. Any product that may be evaluated in this article, or claim that may be made by its manufacturer, is not guaranteed or endorsed by the publisher.
