# Supplementary material for: A rapid turnaround gene panel for severe autoinflammation: Genetic results within 48 hours
Source: Front Immunol. 2022 Sep 20;13:998967. doi: 10.3389/fimmu.2022.998967 (PMC9531256; doi:10.3389/fimmu.2022.998967)
Supplement: Supplementary file 1 [file Table_1.docx]

**Supplemental Table 1:** Disease control genotypes used for validating the RAP. Abbreviations: hom, homozygous; het, heterozygous.

| Control number | | Sample type | Gene | Nucleotide change | Amino acid change | Zygosity |
| --- | --- | --- | --- | --- | --- | --- |
| 1 | Blood | | *ADA2* | c.G139A | p.G47R | Hom |
| 2 | Blood | | *ADA2* | c.G139A | p.G47R | Hom |
| 3 | Blood | | *ADA2* | c.C752T | p.P251L | Het |
| 4 | Blood | | *ADA2* | c.C752T | p.P251L | Het |
| 5 | Blood | | *IFIH1* | c.G2524A | p.E842K | Het |
| 6 | Blood | | *PRF1* | c.C272T | p.A91V | Het |
| 7 | Blood | | *NLRC4* | c.G2785T | p.A929S | Het |
| 8 | Blood | | *LYST* | c.G8806A | p.V2936I | Het |
| 8 | Blood | | *IFIH1* | c.C2138G | p.T713S | Het |
| 9 | Blood | | *IFIH1* | c.A2767G | p.I923V | Het |
| 10 | Blood | | *XIAP* | c.A1268C | p.Q423P | Het |
| 11 | Blood | | *PRF1* | c.1621delA | p.M541Cfs*71 | Het |
| 11 | Blood | | *PRF1* | c.C82T | p.R28C | Het |
| 12 | Blood | | *LYST* | c.T5518G | p.S1840A | Het |
| 13 | Blood | | *LYST* | c.C6781T | p.R2261C | Het |
| 14 | Saliva | | *STING* | c.G659A | p.R220H | Het |
| 15 | Blood | | *-* | - | - | - |
